# Amino acid residues 655 and 969 in the spike protein of Omicron subvariant BA.1 control use of TMPRSS2 versus Cathepsin L dependent entry pathways and cell tropism

**DOI:** 10.1371/journal.pone.0328879

**Published:** 2025-08-14

**Authors:** Cheila Rocha, Prerna Arora, Lu Zhang, Anzhalika Sidarovich, Luise Graichen, Anna-Sophie Moldenhauer, Stefan Pöhlmann, Markus Hoffmann

**Affiliations:** 1 Infection Biology Unit, German Primate Center – Leibniz Institute for Primate Research, Göttingen, Germany; 2 Faculty of Biology and Psychology, Georg-August-University Göttingen, Göttingen, Germany; Emory University, UNITED STATES OF AMERICA

## Abstract

The spike (S) protein of the severe acute respiratory syndrome coronavirus 2 (SARS-CoV-2) is activated by the host cell proteases cathepsin L or TMPRSS2. The ancestral virus circulating in Wuhan in 2020 and early variants mainly use TMPRSS2 for entry into Calu-3 lung cells while the Omicron subvariant BA.1 and most subsequently circulating Omicron subvariants employ both cathepsin L and TMPRSS2 for Calu-3 cell entry. Here, we investigated which amino acid residues in the S protein of the Omicron subvariant BA.1 control protease choice. We show that Y655 promotes S protein cleavage and cathepsin L-dependent entry while H655 jointly with N969 promotes TMPRSS2-dependent entry. These results define molecular signatures of SARS-CoV-2 protease choice and lung cell infection.

## Introduction

Coronavirus disease 2019 (COVID-19), caused by severe acute respiratory syndrome coronavirus 2 (SARS-CoV-2), continues to challenge healthcare systems globally, largely due to the ongoing emergence of new viral variants with enhanced ability to evade immune responses [1–4]. The spike (S) protein, located on the surface of the virus, mediates host cell entry and represents the principal target of neutralizing antibody responses [5]. Understanding how the S protein drives viral entry might define novel targets for antiviral intervention and is in the focus of several ongoing research efforts.

Entry encompasses binding of the S protein to the cellular receptor angiotensin-converting enzyme 2 (ACE2) and fusion of the viral membrane with a target cell membrane, which allows for delivery of the viral genome into the host cell cytoplasm [5,6]. Receptor binding and membrane fusion are driven by different subunits of the S protein. The surface unit, S1, contains a receptor binding domain (RBD) that binds to ACE2 [7] while the transmembrane subunit, S2, harbors a fusion peptide and heptad repeats, which are required for the membrane merger [8,9]. Importantly, the S protein is initially synthesized as an inactive precursor protein and transits into an active state upon cleavage by host cell proteases, which is termed priming or, for historic reasons, activation [10]. Activation of the S protein is essential for SARS-CoV-2 infectivity and the responsible proteases are potential targets for intervention.

The SARS-CoV-2 S protein (SARS-2-S) can employ the cell surface located serine protease TMPRSS2 or the pH-dependent endosomal cysteine protease cathepsin L for activation [5]. Several lines of evidence indicate that TMPRSS2 usage is required for efficient lung cell entry and spread [11–20]. Consequently, the virus circulating in Wuhan at the beginning of the pandemic and most subsequent variants enter Calu-3 lung cells in a TMPRSS2-dependent manner [13,21]. In contrast, the Omicron variant, which became globally dominant at the end of 2021, and most of its subvariants employ both cathepsin L and TMPRSS2 for Calu-3 lung cell entry and, generally, show increased cathepsin L dependence for entry into cell lines [22–25]. Nevertheless, entry of the Omicron variant into primary lung cells is largely TMPRSS2-dependent [20,26] and the Omicron variant exhibits marked but not exclusive TMPRSS2-dependence in mice [13,14]. Notably, with the exception of the Omicron subvariants BA.5 and BA.2.86 [27,28], lung cell entry of the Omicron variant is reduced as compared to previously circulating variants, likely due to less efficient TMPRSS2 engagement, and this may contribute to the diminished lung infection and pathogenic potential of the Omicron variant [22–25]. However, the genetic determinants of protease choice of the Omicron variant remain incompletely understood.

Here, we show that mutation H655Y, which is naturally present in the S protein of the Omicron subvariant BA.1, promotes cathepsin L-dependent entry while introducing mutations Y655H and K969N is sufficient to promote TMPRSS2-dependent entry.

## Materials and methods

### Cell cultures

293T (human kidney, female), Vero (African green monkey kidney, female, kindly provided by Andrea Maisner), Huh-7 cells (human liver, male, kindly provided by Thomas Pietschmann), were maintained in Dulbecco’s modified Eagle medium (DMEM, PAN-Biotech). Calu-3 (human lung, male, kindly provided by Stephan Ludwig) and Caco-2 cells (human colon, male) were maintained in minimum essential medium (GIBCO). Calu-3 cells stably expressing cathepsin L (CTSL, Gene bank NM_001382757) (Calu-3-CTSL) were generated using retroviral transduction and cultured in MEM medium [29]. A549-ACE2 cells [21] which were derived from parental A549 cells (human lung, male, kindly provided by Georg Herrler) were maintained in DMEM/F-12 medium (GIBCO). All media were supplemented with 10% fetal bovine serum (Biochrom) and 100 U/ml penicillin and 0.1 mg/ml streptomycin (PAA). Furthermore, Calu-3 and Caco-2 cells received 1x non-essential amino acid solution (from 100x stock, PAA) and 1 mM sodium pyruvate (GIBCO). Culture medium for A549-ACE2 and Calu-3-CTSL cells was supplemented with 1 μg/ml puromycin. All cell lines were incubated at 37 °C in a humidified atmosphere containing 5% CO_2_. Cell line validation was performed through microscopic examination, partial sequence determination of the cytochrome c oxidase gene, and/or short tandem repeat analysis. All cell lines were regularly tested for absence of contamination by mycoplasma. Transfection of cells was carried out by either calcium-phosphate precipitation, or using Lipofectamine 2000 or Lipofectamine LTX with Plus Reagent (both Thermo Fisher Scientific).

### Plasmids

Plasmids encoding VSV-G (vesicular stomatitis virus glycoprotein), SARS-CoV-2 S B.1 (codon optimized, contains C-terminal truncation of the last 18 amino acid) [5], SARS-CoV-2 S A.30 [30], SARS-CoV-2 S C.1.2 [31], SARS-CoV-2 S P.1 [21] and SARS-CoV-2 S BA.1 [32] have been previously described. Mutants of SARS-CoV-2 variants B.1, A.30, C.1.2, P.1 and BA.1 were generated using overlap extension PCR with overlapping primers harboring the desired mutations (primer sequences are available upon request). Subsequently, the open reading frames were inserted into the pCG1 plasmid (kindly provided by Roberto Cattaneo) using restriction sites BamHI and XbaI. All S protein sequences and the underlying information (collection date, location) were obtained from the GISAID (global initiative on sharing all influenza data) database (https://www.gisaid.org/). The integrity of all PCR amplified sequences was confirmed by automated sequence analysis (Microsynth SeqLab).

### Cell-cell fusion assay

293T effector cells grown to ~75% confluency in 12-well plates were cotransfected with expression plasmids for the respective S protein or empty vector (1.5 µg/well) and the ß-galactosidase α-fragment (0.5 µg/well) using Lipofectamine 2000 (Thermo Fisher Scientific) according to manufacturer’s instructions. In addition, 293T target cells grown to ~75% confluency in 96-well plates were transfected with expression vectors for ACE2 (0.25 µg/well) and the ß-galactosidase omega fragment (0.25 µg/well) using Lipofectamine 2000 (Thermo Fisher Scientific) according to manufacturer’s instructions. The culture medium was changed after 8 h. At 24 h posttransfection, effector cells were washed with PBS and resuspended in 450 µl culture medium, while the culture medium of the target cells was removed. Then, 100 µl effector cell suspension was added to the target cells in technical quadruplicates and cells were incubated for an additional 24 h. Next, ß-galactosidase substrate (Gal-Screen, Thermo Fisher Scientific) was added (100 µl/well) and samples were incubated for 90 min in the dark at room temperature before samples were transferred into white 96-well plates and luminescence was recorded using a Hidex Sense plate luminometer (Hidex).

### Production of VSV pseudoparticles (VSV_pp_)

We generated VSV pseudoparticles (VSV_pp_) as described previously [5,33]. Briefly, 293T cells were transfected with expression plasmid for WT or mutant SARS-CoV-2 S or plasmid encoding DsRed (negative control). At 24 h posttransfection, cells were inoculated with VSV*ΔG FLuc [34] (kindly provided by Gert Zimmer) for 1 h at 37 °C. Next, the inoculum was removed, the cells were washed once with PBS, and DMEM medium containing an anti-VSV-G antibody (produced in I1 hybridoma cells, ATCC CRL-2700) was added to all cells except for those transfected with VSV-G expression vector (these cells received medium without antibody). The cells were further incubated for 24 h before the VSVpp-containing supernatant was harvested, clarified by centrifugation at 4,000 × g for 5 min, and either used directly or stored at −80 °C.

### Transduction of target cells

For transduction experiments, target cells were seeded in 96-well plates 24 h prior to transduction. For transduction, the culture medium was aspirated, and equal volumes of VSV pseudotypes were added to the cells. At 16–18 h posttransduction, transduction efficiency was quantified by measuring the virus encoded firefly luciferase activity in cell lysates using a commercial kit (Beetle-Juice; PJK) and a Hidex Sense plate luminometer (Hidex). Depending on the experimental set-up, target cells were pre-incubated with protease inhibitors (MDL28170 and/or Camostat, 1 h at 37°C) before inoculation with pseudoviruses.

### Immunoblot

To investigate S protein cleavage and particle incorporation, pseudotype particles bearing SARS-CoV-2 S proteins were loaded onto a sucrose cushion (20% w/v sucrose in PBS) and concentrated by high-speed centrifugation (13,300 rpm, 90 min, 4 °C). Following removal of the supernatant, pseudotype particles were lysed in 2 × Sample buffer (0.03 M Tris-HCl, 10% glycerol, 2% SDS, 5% ß-mercaptoethanol, 0.2% bromophenol blue, 1 mM EDTA) and subjected to SDS-PAGE. Next, proteins were transferred onto nitrocellulose membranes (Hartenstein, Würzburg, Germany) using the Mini Trans-Blot Cell system (Bio-Rad, Hercules, CA, USA). Membranes were blocked by incubation in PBS-T (PBS containing 0.05% Tween-20) supplemented with 5% BSA for 30 min. After blocking, membranes were probed overnight at 4 °C with primary antibodies to S2 (1:2000 in PBS-T containing 5% BSA, rabbit, Biozol, Eching, Germany) or VSV-M (1:1000 in PBS-T containing 5% skim milk powder, mouse, Kerafast, Boston, MA, USA). The next day, membranes were washed three times with PBS-T for 10 min and probed for 1h at room temperature with horseradish peroxidase-conjugated secondary antibodies (both 1:2000 in PBS-T containing 5% skim milk powder, Dianova, Hamburg, Germany). Thereafter, membranes were washed three times with PBS-T. Membranes were developed using a homemade chemiluminescence solution (0.1 M Tris-HCl [pH 8.6], 250 g/mL luminol, 0.1 mg/mL para-hydroxycoumaric acid, and 0.3 percent hydrogen peroxide). For imaging, a ChemoCam imager equipped with the ChemoStar Professional software was used (Intas Science Imaging Instruments, Göttingen, Germany). The quantification of protein bands was performed using the ImageJ software (version 1.53C, National Institutes of Health, Bethesda, MD, USA; available at https://imagej.nih.gov/ij/, accessed on 1 October 2022). For the analysis of S protein incorporation into VSV particles, total S protein signals (uncleaved, S0, and cleaved, S2) were normalized against their corresponding VSV-M signals. The resulting values were then compared using the B.1 S protein as a reference (set as 1). In order to quantify S protein cleavage, total S protein signals (uncleaved, S0, and cleaved, S2) were set to 100% for each S protein, and the relative amounts of S0 and S2 were calculated.

### Protein models

Protein models were generated based on PDB: 7–4 [35] using UCSF ChimeraX (version 1.3rc202111192158, University of California, San Francisco, CA, USA; https://www.cgl.ucsf.edu/chimerax/) [36].

### Statistical analysis

Data were analyzed using Microsoft Excel (as part of the Microsoft Office software package, version 2019, Microsoft Corporation) and GraphPad Prism 8 version 8.4.3 (GraphPad Software). Statistical significance was analyzed by unpaired Student’s t-test with Welch correction (pseudotype entry, cell-cell fusion) and by paired Student’s t-test (cleavage efficiency). Statistical significance among multiple groups was analyzed by two-way analysis of variance (ANOVA) with Sidak’s multiple comparison test (concentration dependent inhibitors). Only p values of 0.05 or lower were considered statistically significant (p > 0.05; not significant [ns], p ≤ 0.05; [*], p ≤ 0.01; [**], p ≤ 0.001; [***]). Details on the statistical test and the error bars can be found in the figure legends.

## Results

### Association of mutation H655Y with cathepsin L-dependent cell entry

**Fig 1:**
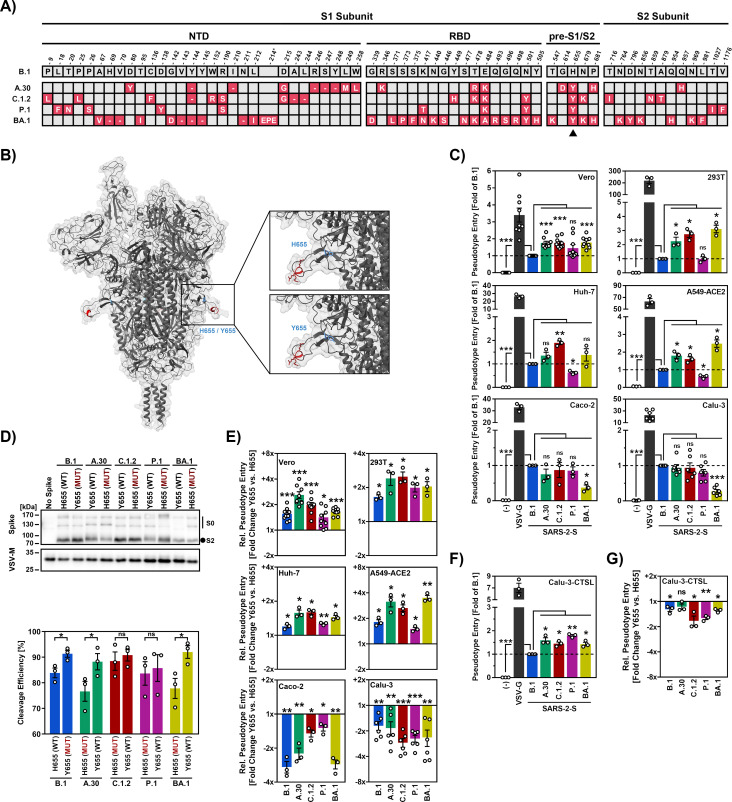
Mutation H655Y in the SARS-CoV-2 S protein promotes S protein cleavage and entry into cell lines previously found to only allow for cathepsin L-dependent entry. (A) Overview of mutated S protein residues in SARS-CoV-2 lineages A.30, C.1.2, P.1 and BA.1 compared to B.1 (numbering according to the B.1 S protein; the BA.1 S protein harbors a three amino acid [EPE] insertion between residues 214 and 215). The shared H655Y mutation by SARS-CoV-2 lineages A.30, C.1.2, P.1 and BA.1 is highlighted by a black arrowhead. Abbreviations: NTD = N-terminal domain, RBD = receptor-binding domain, pre-S1/S2 = region between the RBD and the S1/S2 cleavage site. (B) 3-dimensional structure of the SARS-CoV-2 S protein trimer in which the S1/S2 cleavage site (red) and the mutation H655Y are highlighted. (C) S protein-driven host cell entry. Particles pseudotyped with the indicated S proteins were inoculated onto cell lines that either allow for entry mainly via the endosomal pathway (Vero, 293T, Huh-7, A549-ACE2) or the cell surface pathway (Caco-2, Calu-3). Particles bearing VSV-G or no viral surface protein were used as positive and negative controls, respectively. Pseudovirus entry was analyzed at 16-18 h postinoculation by measuring the activity of virus-encoded luciferase in cell lysates. Presented are the mean data of three to nine biological replicates (each conducted with four technical replicates) for which entry was normalized against B.1 (set as 1). Error bars indicate the standard error of the mean (SEM). (D) Augmented S protein cleavage due to mutation H655Y. Top: Pseudovirus particles bearing the indicated S proteins harboring either H655 or Y655 were subjected to SDS-PAGE and S protein cleavage was analyzed by immunoblot using antibodies directed against the S2 subunit of SARS-CoV-2 S protein and VSV-M (loading control). Pseudovirus particles bearing no S protein served as control. A representative immunoblot is shown and similar results were obtained in two separate experiments. Bands indicating the uncleaved S0 precursor S protein and the S2 subunit of the cleaved S protein are highlighted. Bottom: Band intensities for uncleaved (S0) and cleaved (S2) S protein were quantified using the ImageJ software and cleavage efficiency (percentage of S2 signals relative to total S protein signals [S2 + S0]) was calculated. Presented are the mean data from three biological replicates with error bars indicating the SEM. (E) Mutation H655Y augments entry via the endosomal pathway while reducing entry via the cell surface pathway. Pseudovirus particles bearing the indicated S proteins harboring either H655 or Y655 were inoculated onto the indicated cell lines and pseudovirus entry was analyzed as described for panel C. Presented are the mean data of three to nine biological replicates (each conducted with four technical replicates). Entry driven by S proteins containing H655 was set as 1 and the fold change in entry efficiency by the corresponding S proteins bearing Y655 was calculated. Error bars indicate the SEM. (F) Overexpression of cathepsin L in Calu-3 cells (Calu-3-CTSL) compensates for reduced cell entry due to S protein mutation H655Y. Pseudovirus particles bearing the indicated S proteins harboring either H655 or Y655 were inoculated onto cathepsin L-overexpressing Calu-3 cells (Calu-3-CTSL) cells. Particles bearing VSV-G or no viral surface protein were used as positive and negative controls, respectively. Pseudovirus entry was analyzed at 16-18 h postinoculation by measuring the activity of virus-encoded luciferase in cell lysates. Presented are the mean data of three biological replicates (each conducted with four technical replicates) for which entry was normalized against B.1 (set as 1). Error bars indicate the SEM. (G) The experiment was conducted as described for panel (E) but Calu-3-CTSL cells were used as targets. Presented are the mean data of three biological replicates (each conducted with four technical replicates) for which driven by S proteins containing H655 was set as 1 and the fold change in entry efficiency by the corresponding S protein bearing Y655 was calculated. Error bars indicate SEM. Statistical analysis: For panels C and E-G statistical significance was analyzed by unpaired Student’s t-test with Welch correction. For panel D, statistical significance was analyzed by paired Student’s t-test (not significant [ns], p > 0.05; *, p ≤ 0.05; **, p ≤ 0.01; ***, p ≤ 0.001).

In order to identify the amino acid residues in the S protein that are responsible for the preference of the Omicron variant for cathepsin L-dependent entry into cell lines, we focused on residue 655 (Fig 1A) since this residue is located at the base of the cleavage loop harboring the furin cleavage site (Fig 1B) and modulates S protein cleavage [37]. H655 is found in SARS-CoV-2 variant B.1, which circulated early in the pandemic, while the S protein of SARS-CoV-2 Omicron subvariant BA.1 harbors mutation H655Y (Fig 1A). Mutation H655Y is also present in the S proteins of the P.1 (Gamma variant), A.30 and C.1.2 variants, and we previously reported that the latter two S proteins exhibit increased cathepsin L-dependent entry [30,31]. Indeed, a systematic analysis revealed augmented Vero, 293T, Huh-7 and A549-ACE2 cell entry, which is known to be cathepsin L dependent [5,11,12,22,38,39], of particles bearing A.30 or C.1.2 S proteins (A.30_pp_, C.1.2_pp_) relative to B.1_pp_ (Fig 1C). In contrast, no marked differences were observed for entry into Caco-2 and Calu-3 cell entry (Fig 1C), which is mainly promoted by TMPRSS2. Entry of P.1_pp_ into Vero, 293T, Huh-7 and A549-ACE2 cells was similar or reduced as compared to B.1_pp_ and again no major differences were observed for Caco-2 and Calu-3 cell entry. Finally, BA.1_pp_ showed, relative to B.1_pp_, augmented Vero, 293T, Huh-7 and A549-ACE2 cell entry but reduced Caco-2 and Calu-3 cell entry (Fig 1C), as expected [40]. Thus, A.30_pp_, C.1.2_pp_ and BA.1_pp_ showed a preference for cathepsin L-dependent entry compared to B.1_pp_ and harbored the mutation H655Y.

### H655Y promotes S protein cleavage and cathepsin L-dependent cell entry

We next investigated whether the differences in cell tropism of the pseudoparticles studied were dependent on amino acid residue 655. Mutagenic analysis demonstrated that mutation H655Y markedly increased cleavage of B.1, A.30 and BA.1 S protein while cleavage efficiency of C.1.2 and P.1 S protein was only slightly augmented (Fig 1D). Further, mutation H655Y increased Vero, 293T, Huh-7 and A549-ACE2 cell entry (cathepsin L-dependent) driven by all S proteins tested and the reverse phenotype was detected for Caco-2 and Calu-3 cell entry (TMPRSS2 dependent) (Fig 1E). Further, overexpression of cathepsin L in Calu-3 cells, which has been demonstrated previously [41], increased host cell entry of A.30_pp_, C.1.2._pp_, P.1_pp_ and BA.1_pp_ relative to B.1_pp_ (Fig 1F). Finally, mutation H655Y only moderately reduced entry into Calu-3 cells stably expressing cathepsin L (Fig 1G) while this effect was prominent for Calu-3 WT cells (Fig 1E). Collectively, mutation H655Y increased cleavage of most S proteins and skewed tropism towards cells that allow for cathepsin L-dependent SARS-CoV-2 entry.

### H655Y reduces susceptibility to entry inhibition by MDL28170, a cathepsin L inhibitor

**Fig 2:**
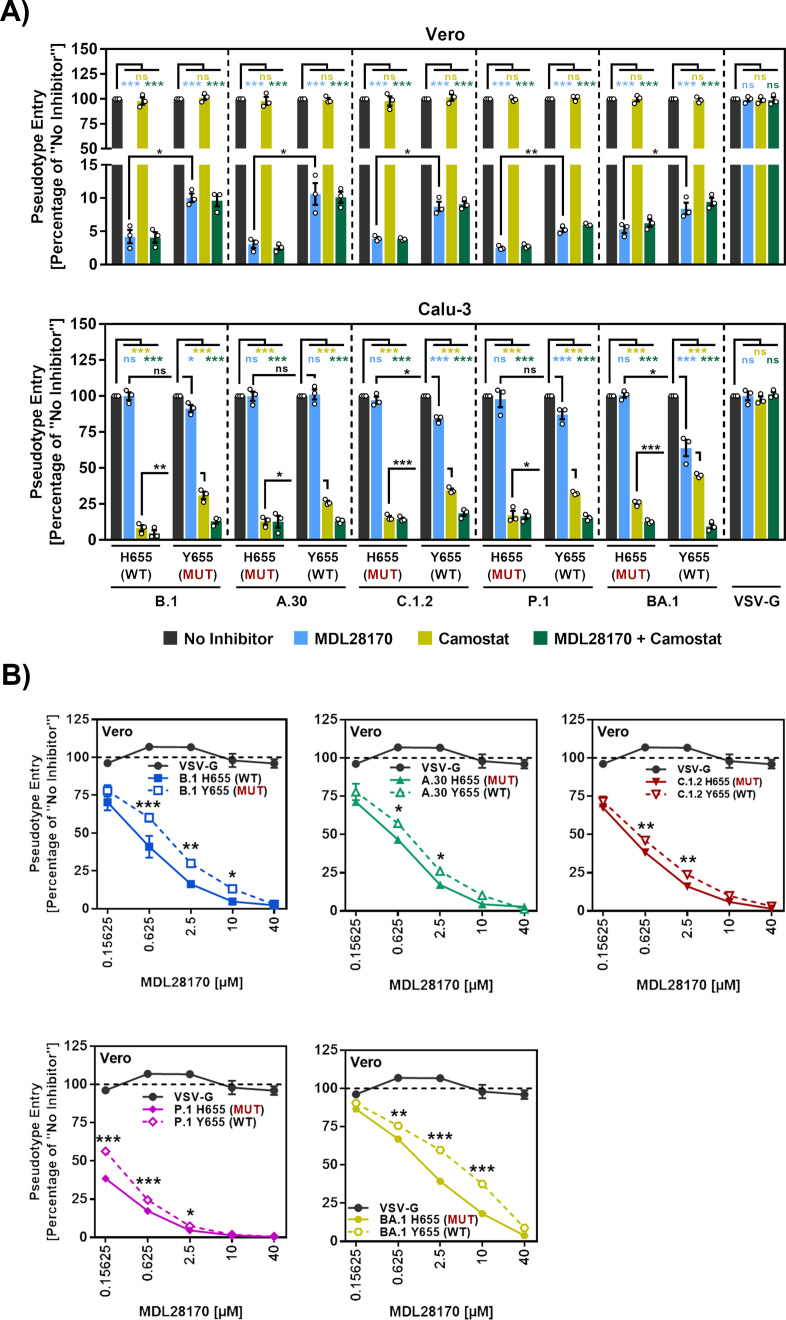
Mutation H655Y in the SARS-CoV-2 S protein promotes entry via the endosomal pathway by increasing cathepsin L usage. (A) Mutation H655Y reduces sensitivity of S protein-driven cell entry to blockade of the endosomal pathway. Vero and Calu-3 cells were preincubated (2 h, 37°C) with medium containing either the cathepsin/calpain inhibitor MDL28170 (25 µM), the TMPRSS2 inhibitor Camostat mesylate (25 µM), a combination of both inhibitors (25 µM/25 µM) or DMSO (no inhibitor control), before pseudovirus particles bearing the indicated S proteins containing either H655 or Y655 were added (pseudoviruses bearing VSV-G served as control). Pseudovirus entry was analyzed at 16-18 h postinoculation by measuring the activity of virus-encoded luciferase in cell lysates. Presented are the mean data of three biological replicates (each conducted with four technical replicates) for which entry in the absence of inhibitor was set as 100%. Error bars indicate SEM. (B) Mutation H655Y increases Vero cell entry under conditions of low cathepsin L availability. Vero cells were preincubated (2 h, 37°C) with medium containing different concentrations of the cathepsin/calpain inhibitor MDL28170 or DMSO (no inhibitor control), before pseudovirus particles bearing the indicated S proteins containing either H655 or Y655 were added (pseudoviruses bearing VSV-G served as control). Pseudovirus entry was analyzed as described for panel A. Presented are the mean data of three biological replicates (each conducted with four technical replicates) for which entry in the absence of inhibitor was set as 100%. Error bars indicate SEM. Statistical analysis: For panel A statistical significance was analyzed by unpaired Student’s t-test with Welch correction. For panel B, statistical significance among multiple groups was analyzed by two-way analysis of variance (ANOVA) with Sidak’s multiple comparison test, (not significant [ns], p > 0.05; *, p ≤ 0.05; **, p ≤ 0.01; ***, p ≤ 0.001).

We next employed the cathepsin L inhibitor MDL28170 and the TMPRSS2 inhibitor Camostat to explore the impact of residue 655 on protease choice. MDL28170 but not Camostat potently inhibited Vero cell entry driven by all S proteins tested and inhibition efficiency was decreased by mutation H655Y (Fig 2A, upper panel), suggesting that H655Y promotes cathepsin L-dependent entry. Conversely, Camostat but not MDL28170 efficiently blocked Calu-3 cell entry driven by all S proteins tested (Fig 2A, lower panel). A notable exception was entry driven by BA.1 S protein, which was sensitive to inhibition by both MDL28170 and Camostat (Fig 2A, lower panel), in keeping with the previously noted preference of Omicron variants for the cathepsin L- versus the TMPRSS2-dependent entry pathway [22–25,40]. Further, mutation H655Y decreased sensitivity to entry inhibition by Camostat (Fig 2A, lower panel), in keeping with increased ability of these S proteins to employ cathepsin L for host cell entry in case TMPRSS2 is not available (Fig 1E and Fig 2A, lower panel). Indeed, residue Y655 decreased susceptibility of all S proteins tested to inhibition by MDL28170 over a range of concentrations (Fig 2B), further supporting the notion that exchange H655Y might increase the ability to use cathepsin L for entry and that this effect might be largely independent of the S protein context.

### Both H655 and Y655 are compatible with robust S protein-driven cell-cell fusion

**Fig 3:**
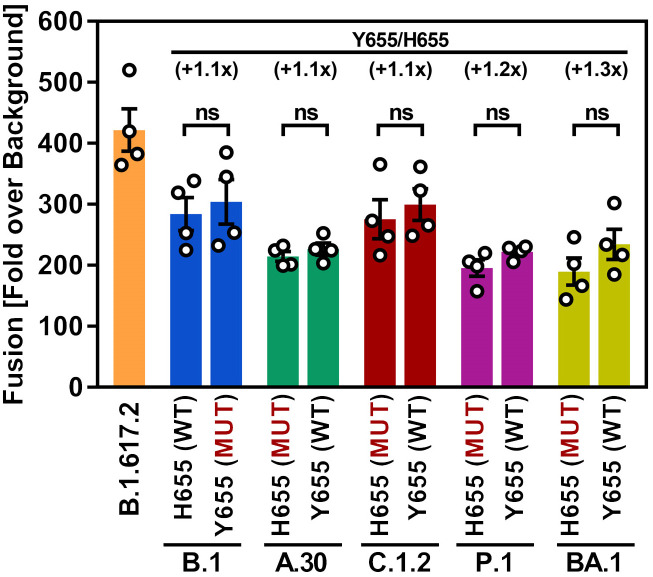
Mutations H655Y or Y665H do not impact S protein-driven cell-cell fusion. 293T effector cells transiently expressing the respective S proteins (or no S protein, control) along with the α-fragment of ß-galactosidase were mixed with 293T target cells transiently expressing ACE2 and the ß-galactosidase omega fragment. At 24 h post mixing, the activity of reconstituted ß-galactosidase in fused effector and target cells was measured as a proxy for cell-cell fusion. Presented are the mean data of four biological replicates (each conducted with three technical replicates) for which ß-galactosidase activity was normalized against the assay background (signals obtained for target cells that were mixed with effector cells that did not express any S protein). Error bars indicate SEM. Statistical significance was analyzed by unpaired Student’s t-test with Welch correction (ns, p > 0.05; *, p ≤ 0.05; **, p ≤ 0.01; ***, p ≤ 0.001).

We next tested the impact of residue 655 on S protein-driven cell-cell fusion. For this, the S proteins of B.1, A.30, C.1.2, P.1 and BA.1 harboring either H655 or Y655 were expressed on effector cells and fusion with ACE2-expressing target cells was analyzed. The S protein of B.1.617.2 (Delta variant) was included as positive controls since it drives cell-cell fusion with increased efficiency due to an additional arginine residue at the S1/S2 cleavage site [42]. The B.1.617.2 S protein drove cell-cell fusion with highest efficiency and the B.1 (WT) S protein enabled cell-cell fusion more efficiently than the BA.1 S protein, as expected (Fig 3) [27,42]. Insertion of Y655 into B.1 S protein did not increase cell-cell fusion and similar findings were made for the A.30 and C.1.2 S proteins. In the context of the P.1 and BA.1 S protein Y655 increased cell-cell fusion but the effect was minor (Fig 3).

### Y655H and K969N restore BA.1 entry into Calu-3 lung cells to B.1 levels

**Fig 4:**
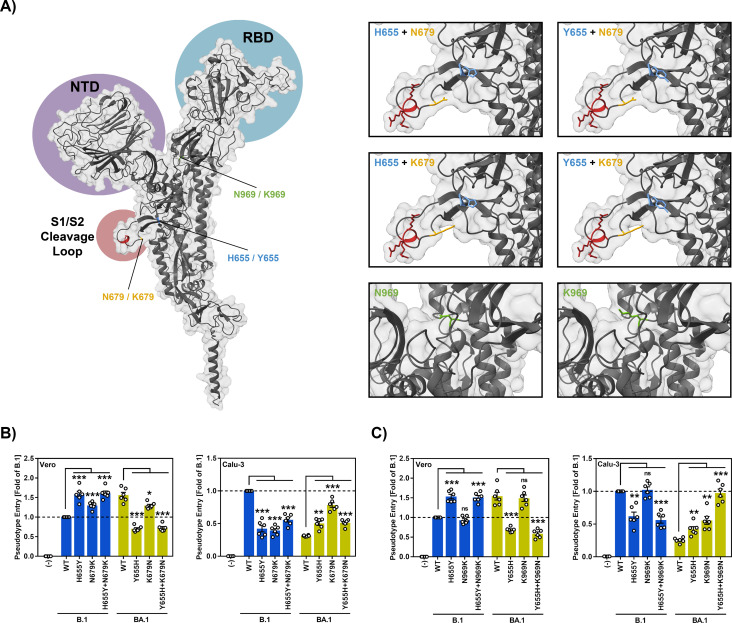
H655 combined with N969 allows for robust TMPRSS2-dependent entry driven by the BA.1 S protein. (A) Left panel: 3-dimensional structure of the SARS-CoV-2 S protein monomer in which the N-terminal domain (NTD, purple), the receptor binding domain (RBD, blue) and the S1/S2 cleavage loop (red) are indicated. Further, the location of the amino acid residues under study is indicated. Right panels: Close-up of the regions encompassing the mutations under study and predicted structural consequences of the mutations. (B) K679N increases Calu-3 entry driven by BA.1 spike. Pseudovirus particles bearing the indicated WT or mutated S proteins were inoculated onto Vero and Calu-3 cells. Particles bearing no viral surface protein were used as negative control. Pseudovirus entry was analyzed at 16-18 h postinoculation by measuring the activity of virus-encoded luciferase in cell lysates. Presented are the mean data of six biological replicates (each conducted with four technical replicates) for which entry was normalized against B.1 (set as 1). Error bars indicate SEM. (C) Combining H655 and N969 increases Calu-3 entry driven by BA.1 spike. The experiment was conducted as described for panel B. Presented are the mean data of six biological replicates (each conducted with four technical replicates) for which entry was normalized against B.1 (set as 1). Error bars indicate SEM. Statistical significance was analyzed by unpaired Student’s t-test with Welch correction (ns, p > 0.05; *, p ≤ 0.05; **, p ≤ 0.01; ***, p ≤ 0.001).

We finally analyzed whether residues other than 655 might impact protease choice. For this, we focused on BA.1 S protein mutations (relative to B.1 S protein) N679K and N969K. Mutation N679K, like H665Y, is located at the base of the cleavage loop while N969K resides in the heptad repeat 1 (HR1) of the S2 subunit (Fig 4A). Both mutations were suggested by two preprints to modulate the entry pathway of the Omicron variant [43,44]. Mutagenic analyses confirmed that Y655 promoted Vero cell entry, which is cathepsin L-dependent, while H655 promoted Calu-3 cell entry, which is TMPRSS2 dependent (Fig 4B). A similar tendency was observed for K679 (favors cathepsin L dependent entry) and N679 (favors TMPRSS2-dependent entry) but combining mutations at positions 655 and 679 did not result in additive effects (Fig 4B). Residue K969 had little impact on Vero and Calu-3 cell entry of B.1_pp_ (Fig 4B). In contrast, N969 promoted Calu-3 but not Vero cell entry of BA.1_pp_ and this effect was augmented when N969 was combined with H655, which in turn reduced Vero cell entry (Fig 4C). These results suggest that while H/Y655 is an important regulator of endosomal versus cell surface entry of BA.1 and other SARS-CoV-2 variants other amino acid residues can impact entry route preference with H655 combined with N969 allowing for robust TMPRSS2-dependent entry driven by the BA.1 S protein.

## Discussion

Published findings, confirmed by the present study, demonstrate that the Omicron variant (BA.1) exhibits a bias toward entering cell lines via the endosomal pathway, which depends on cathepsin L activity, rather than utilizing the TMPRSS2-dependent entry route at the cell surface [22,40]. Furthermore, we demonstrate that mutation Y655 directs entry driven by BA.1 S protein and diverse other S proteins to the endosomal route while H655 promotes entry via the cell surface route, resulting in increased entry into Calu-3 lung cells, which express endogenous TMPRSS2. In contrast, this mutation had only a moderate impact on S protein-driven cell-cell fusion. Finally, we show that K679 alone or K969 in conjunction with Y655 also promote BA.1 entry via the cathepsin L-dependent route while N679 and N969 jointly with H655 have the opposite effect, indicating that several amino acid residues control protease choice of the BA.1 S protein.

We found that Y655 was compatible with robust cleavage or even increased cleavage efficiency, in keeping with several published studies or preprints demonstrating that exchange Y655H in BA.1 S protein either decreased [45] or had no effect on cleavage [46] while H655Y was found to either not affect or increase cleavage in the context of WT S protein [37,47,48]. The efficiency of S protein cleavage at the S1/S2 site correlates with the efficiency of S protein-driven cell-cell fusion. We found, in keeping with several previous studies, that B.1 S protein facilitated cell-cell fusion with higher efficiency than BA.1 S protein and that cell-cell fusion driven by the B.1.617.2 (Delta variant) S protein was most efficient, due to the presence of an optimized cleavage motif at the B.1.617.2 S1/S2 site [25,40,42,49]. We note that Y655H slightly decreased cell-cell fusion by BA.1 and P.1 S proteins but neither H655 nor Y655 modulated cell-cell fusion driven by B.1, C.1.2 and A30 S proteins. These results indicate that residue 655 might have no or a minor role in cell-cell fusion. Hu and colleagues as well as Park and coworkers found that H655Y decreased fusion driven by B.1 S protein while others reported that this exchange increased fusogenicity of WAI S protein (which is closely related to the B.1 S protein) [37]. Furthermore, other studies documented that mutation Y655H augments cell-cell fusion in the context of the S protein of the Omicron variant [48,50]. The reason for the discrepancies between these results and the data reported here are at present unclear but might be related to S protein expression levels or the cellular system chosen for analysis of S protein-driven cell-cell fusion. Thus, the role of residue 655 in cell-cell fusion requires further analyses.

Our analyses of S protein-mediated cell entry indicated a correlation between the presence of Y655 in the S protein of SARS-CoV-2 variants B.1, A.30, C.1.2, P.1 and BA.1 and increased entry into cell lines that only offer the cathepsin L-dependent entry route while the presence of H655 was associated with increased entry into cell lines expressing TMPRSS2. Importantly, this finding was fully supported by mutagenic analyses formally demonstrating that Y655 promotes cathepsin L- while H655 augments TMPRSS2-dependent entry and other studies reported comparable results [45–47,50]. However, residues other than H/Y655 can modulate entry route preference and/or cell tropism. Thus, mutation N679K increased Vero- but decreased Calu-3 cell entry of B.1_pp_ and a separate study reported that this mutation decreased S protein incorporation into particles and infection of cell lines [44]. However, the mutation augmented viral spread in the upper respiratory tract in a rodent model and thus likely increases transmissibility [44]. One can speculate that increased spread of mutant N679K in the upper respiratory tract, a hallmark of the Omicron variant, might be due to increased cathepsin L-usage, as indicated by the augmented Vero cell entry observed in the present study. Mutation N969K is located in HR1 and was reported to alter the structure of HR2 within the HR1-HR2 six-helix-bundle [51], which is associated with completed membrane fusion, although the mutation increases intra-protomer hydrophobic packing in the context of the prefusion conformation [52]. Furthermore, mutation N969K in WT S protein was reported to reduce infection [53]. In the present study, N969K had little impact on entry of B.1_pp_ into Vero and Calu-3 cells while the reverse exchange increased BA.1_pp_ entry into both Vero and Calu-3 cells. Furthermore, combination of Y655H and K969N markedly promoted Calu-3 cell entry of BA.1_pp_. It is at present unclear why differential effects of N969K on infectivity of particles bearing WT S protein were observed in the previous and the present study but differences in the target cells used (Caco-2 [53] versus Vero/Calu-3 (present study)) might be responsible. Nevertheless, the results available at present suggest that besides H655Y also mutations N679K and N969K can contribute to the Omicron specific entry route preferences and cell tropism.

## Conclusion

The Omicron variant of SARS-CoV-2 shows, unlike all previously circulating variants, a preference for cathepsin L- over TMPRSS2-dependent entry into cell lines. The present study, jointly with previous work, identifies amino acid position 655 in the Omicron S protein as a molecular switch between cathepsin L- and TMPRSS2-dependent entry. Thus, Y655 promotes cathepsin L-dependent entry while H655 increases TMPRSS2-dependent entry and the latter is further augmented by N969. However, augmented cathepsin L-dependence linked to Y665 was demonstrated for the S proteins of Omicron subvariant BA.1 and pre-Omicron variants. Therefore, it remains to be determined whether this residue, which is also present (jointly with K969) in various other Omicron subvariants including JN.1 and KP.3.1.1, impacts protease choice in the context of these S proteins.

## Supporting information

S1 Table Data sets used to generate the graphs. (XLSX)

S1 Fig Unprocessed western blot image (Spike, corresponding to figure 1D, upper panel). (TIF)

S2 Fig Unprocessed western blot image (VSV-M, corresponding to figure 1D, lower panel). (TIF)
