# Supplementary figures and images for: Amino acid residues 655 and 969 in the spike protein of Omicron subvariant BA.1 control use of TMPRSS2 versus Cathepsin L dependent entry pathways and cell tropism
Source: PLoS One. 2025 Aug 14;20(8):e0328879. doi: 10.1371/journal.pone.0328879 (PMC12352760; doi:10.1371/journal.pone.0328879)

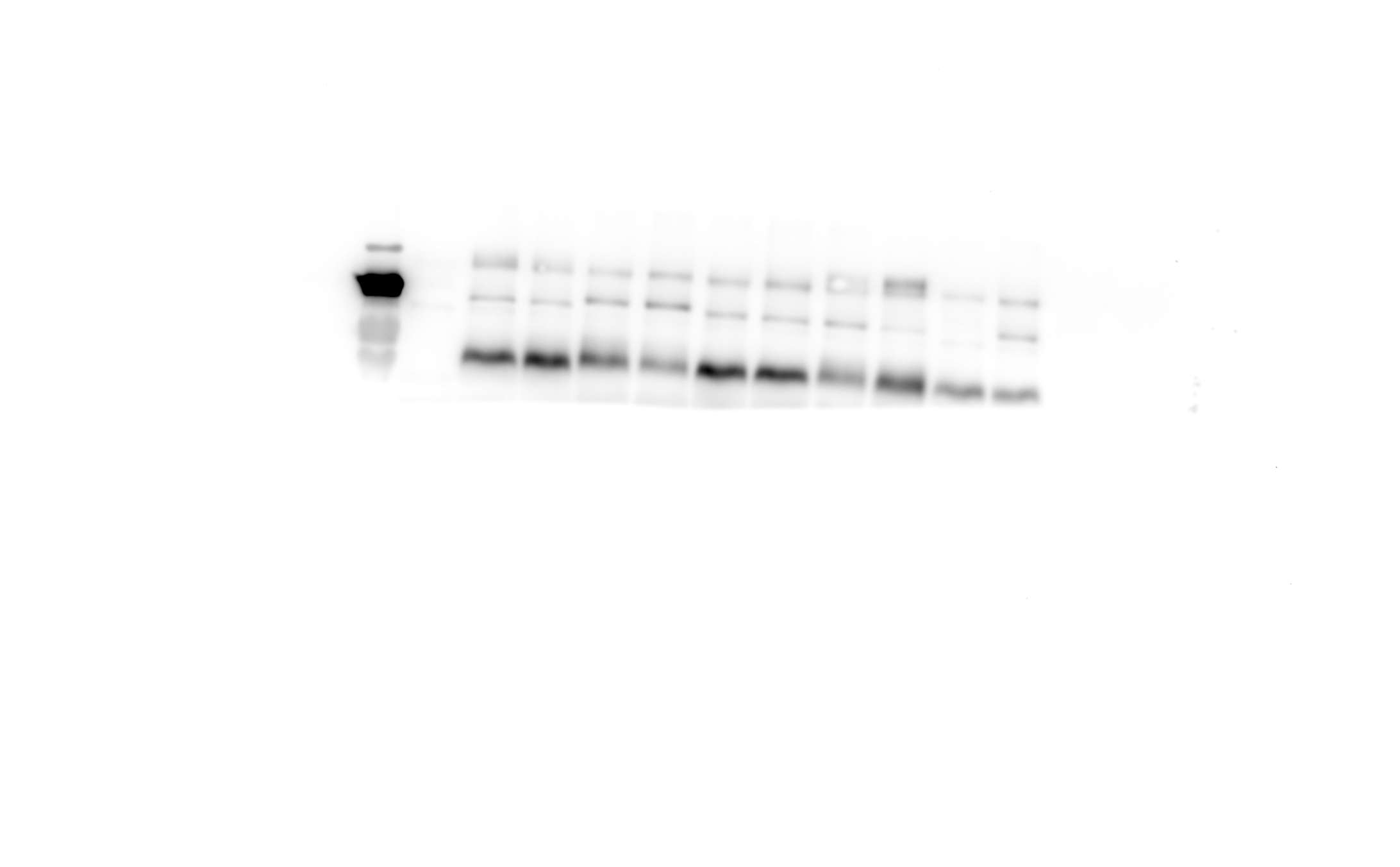

Supplement: S1 Fig — (TIF) [file pone.0328879.s002.tif]

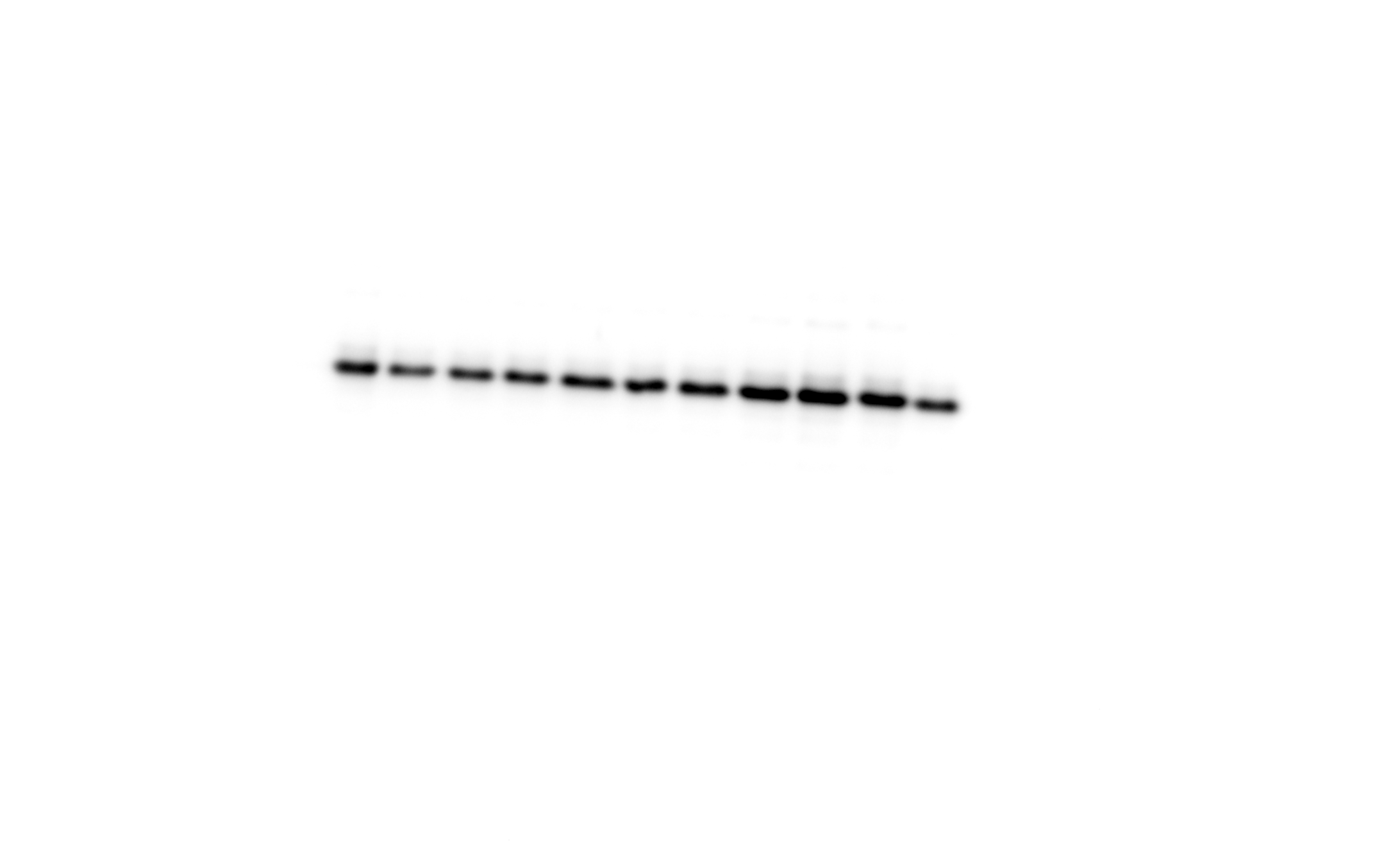

Supplement: S2 Fig — (TIF) [file pone.0328879.s003.tif]
